# Acute Effects of Vardenafil on Pulmonary Artery Responsiveness in Pulmonary Hypertension

**DOI:** 10.1100/2012/718279

**Published:** 2012-05-02

**Authors:** Edibe Karasu-Minareci, Irem Hicran Ozbudak, Gulay Ozbilim, Gulay Sadan

**Affiliations:** ^1^Department of Pharmacology, Akdeniz University School of Medicine, 07070 Antalya, Turkey; ^2^Department of Pathology, Akdeniz University School of Medicine, 07070 Antalya, Turkey

## Abstract

Phosphodiesterase type-5 (PDE-5) inhibitors are novel and important options for the treatment of pulmonary arterial hypertension (PAH). Therefore, we aimed to examine effects of vardenafil, a PDE-5 inhibitor, on the pulmonary arteries isolated from rats with monocrotaline- (MCT-) induced pulmonary hypertension. MCT (60 mg/kg) or its vehicle was administered by a single intraperitoneal injection to 6-week-old male Sprague Dawley rats. Rats were sacrificed 21 days after MCT injection, and the main pulmonary arteries were isolated and then mounted in 20 mL organ baths. Concentration-response curves for vardenafil (10^−10^–10^−5^ M) were constructed in phenylephrine- (Phe-) precontracted rings. PAH caused marked rightward shift in the curves to vardenafil whereas maximal responses were not affected. Inhibition of NO synthase (L-NAME, 10^−4^ M) or guanylyl cyclase (ODQ, 10^−5^ M) caused similar attenuation in responses evoked by vardenafil. Moreover, contraction responses induced by CaCl_2_ (3×10^−5^–3×10^−2^ M) were significantly reduced in concentration-dependent manner by vardenafil. In conclusion, vardenafil induced pulmonary vasodilatation via inhibition of extracellular calcium entry in addition to NO-cGMP pathway activation. These results provide evidence that impaired arterial relaxation in PAH can be prevented by vardenafil. Thus, vardenafil represents a valuable therapeutic approach in PAH besides other PDE-5 inhibitors.

## 1. Introduction

Pulmonary arterial hypertension (PAH) is a severe disease with a poor prognosis. In untreated patients, there is a progressive increase in pulmonary vascular resistance that leads to intractable right ventricular failure and premature death [1]. The median period of survival after diagnosis is about 2-3 years [2]. Despite recent major improvements in symptomatic treatments, no current treatment cures this devastating condition [3]. Therefore, a novel therapeutic strategy for pulmonary hypertension is desirable.

The pathogenesis of PAH is multifactorial [4]. Besides vasoconstriction, endothelial cell dysfunction was also thought to play integral role in the pathogenesis of PAH [5]. This endothelial dysfunction is characterized by an overproduction of vasoconstrictors, proliferative factors, such as endothelin-1, and a reduction of vasodilators, antiproliferative factors, such as prostacyclin and nitric oxide (NO). The potent vasodilator and antiproliferative activity of NO is mediated via its second messenger, cyclic guanosine monophosphate (cGMP), in pulmonary system [6]. Intracellular cGMP is rapidly inactivated to GMP by the activity of cyclic nucleotide phosphodiesterases (PDEs) [7]. Inhibition of the cGMP-specific phosphodiesterase type 5 (PDE-5) leads to an accumulation of cGMP enhancing the action of NO. Within the pulmonary circulation, PDE-5 is the most abundantly expressed isoform and appears to be upregulated in PAH [8–10]. Moreover, Preston et al. reported an interesting clinical study of patients with acute and chronic pulmonary hypertension in which the specific PDE-5 inhibitor, sildenafil, is a potent acute pulmonary vasodilator and addition of inhaled nitric oxide potentiates these effects [11]. Also they concluded that sildenafil is well tolerated in these patients. Recently, another PDE-5 inhibitor, tadalafil, was also granted regulatory approval on the basis of the demonstration of favorable effects on exercise capacity and quality of life and improvements in time to clinical worsening [12].

To the best of our knowledge, studies investigating the efficacy of vardenafil to treat PAH are still limited to few case reports and clinic studies [13, 14]. This specific PDE-5 inhibitor has not yet undergone an extensive in vitro pharmalogical study in pulmonary hypertension model. Therefore, we aimed to explore the effects of vardenafil in monocrotaline-induced pulmonary hypertension and investigate the underlying mechanisms in these effects.

## 2. Material and Methods

This study was approved by the Animal Ethics Committee of Akdeniz University Medical Faculty.

### 2.1. Drugs

Vardenafil was provided by Bayer Health Care AG, Leverkusen, Germany. Acetylcholine (Ach), ethylene glycol tetraacetic acid (a chelator agent for calcium ion, EGTA), *N* ^*ω*^-nitro-L-arginine methyl ester (nitric oxide synthase inhibitor, L-NAME), 1*H*-[1,2, 4]oxadiazolo [4,3, -*a*]quinoxalin-1-one (guanylyl cyclase inhibitor, ODQ), phenylephrine (Phe), and monocrotaline (MCT) were purchased from Sigma-Aldrich (St. Louis, MO). Stock solutions were prepared in deionized water, except vardenafil and ODQ, which were prepared in dimethyl sulfoxide and stored in aliquots at −20°C; dilutions were made in deionized water immediately before use. The final concentration of dimethyl sulfoxide did not exceed 0.1%. Preliminary experiments ascertained the lack of response to either vehicle in the concentrations employed.

### 2.2. Monocrotaline-Induced Pulmonary Hypertension Model

Monocrotaline is a member of the alkaloid family of plant toxins that induces a delayed, yet progressive vascular injury resulting in pulmonary hypertension in rats, dogs, and monkeys [15, 16]. It is recognized that the initial reaction to MCT-induced pulmonary hypertension is injury to the endothelial cells that precedes media hypertrophy in small-size pulmonary arteries and leads to an increase in pulmonary artery disease. The MCT-induced rat model has been widely used as an experimental model of pulmonary hypertension [17].

MCT was dissolved in 1.8 mL 1 M HCl, and 3-4 mL of distilled water was added, as described previously [18]. This solution was adjusted to pH 7.4 with 1 M NaOH and filled up to 15 mL by distilled water. The animals were divided into 2 groups: group 1 received vehicle solution (as control group, *n*: 20); group 2 (*n*: 20) received monocrotaline. MCT (60 mg/kg) or its vehicle was administered to 6-week-old male Sprague Dawley rats (180–210 g) as a single intraperitoneal injection. Rats were housed with a 12 : 12 light-dark cycle and given water and standard rat chow ad libitum. All experiments were carried out 21 days after the administration.

### 2.3. Tissue Preparations

After 21 days, each animal was anesthetized with pentobarbital (45 mg/kg). The heart and lungs were removed by en bloc resection. Right and left main pulmonary arteries were dissected and harvested for vascular reactivity. After removal of the arteries, right ventricle (RV) and left ventricle with septum (LV + S) were separated and weighed. Also in some animals both from control (*n*: 15) and MCT-treated (*n*: 16) groups, the right ventricle, left ventricle, main pulmonary arteries, and the inferior lobes of the lungs fixed in 10% buffered formalin embedded in paraffin for light microscopy and histopathological examinations.

### 2.4. Histopathological Examination

Left and right lungs of rats were removed from the thoracic cavity, and histological specimens were fixed overnight by instillation of 10% buffered formalin into the airways and vascular structures. And then, representative cross-sections of the lungs which include the peripheral and the central pulmonary arteries were sampled and embedded in paraffin blocks. Serial sections (5-*μ*m-thick) were prepared and stained with hematoxylin and eosin for the assessment of vascular pathology. Pulmonary arteries were identified as vessels which had two clearly defined elastic laminas. SAMBA TPS Version 5.04, 1999 was used to assess the pulmonary arteries by the same pathologist, from Akdeniz University School of Medicine Department of Pathology. This observer was unaware of the groups. The percent wall thickness (WT %) of arteries (diameter; 15–150 *μ*m) was calculated by using the following formula as described previously [19, 20]: WT% = 2 × WT/external diameter (ED) × 100. The thickness of medial wall (WT) was measured under the microscope as the distance between the external and internal elastic lamina, as seen with the use of a calibrated eyepiece. ED was measured as the diameter of external lamina. For each rat, *∼*20–25 vessels were counted, and an average was calculated. The vessels that were close to round or oval in shape were measured. Medial WT and the ED measurements were made at several points of each vessel, and an average was calculated.

### 2.5. Vascular Reactivity Studies

Experiments were conducted on isolated main left and right pulmonary arteries. Main pulmonary artery branches were rapidly removed, gently cleaned of fat and connective tissues taking care not to damage the endothelium and cut into rings of about 3 mm then carefully suspended by two stainless-steel clips passed through the vessel lumen in a 20 mL organ bath containing Krebs solution (in mM: NaCl 136.9, KCl 5.4, CaCl_2_ 1.5, MgCl_2_ 1.0 and glucose 5.5.) at 37°C, and was continuously aerated with 95% O_2_ and 5% CO_2_ to obtain a pH of 7.4. Isometric tension was continuously measured with an isometric force transducer (FDT-05 Force Displacement Transducer, BioPac), connected to a computer-based data acquisition system (MP30 Transducer Data Acquisition System, BioPac). The tissue was equilibrated for 60 min under a resting tension of 1 g. During this time, Krebs solution was replaced every 15 minutes with fresh solution. After the 1 hour equilibration period, pulmonary rings were challenged with 80 mM KCl (the same composition as Krebs' solution with NaCl replaced by equimolar KCl) to check tissue viability. Next, the endothelial integrity of the preparations was determined by verifying the responsiveness to ACh (10^−6^ M) in vessels precontracted with Phe (10^−6^ M). Rings were then washed several times to restore tension to the baseline level.

In the first set of experiments, concentration-response curves (10^−10^–10^−5^ M) for vardenafil were constructed in precontracted (Phe, 10^−6^ M) control or pulmonary hypertensive rat pulmonary artery rings in the absence or in the presence of L-NAME (10^−4^ M) or ODQ (10^−5^ M). One concentration-response curve to vardenafil was obtained in each segment. Hence, control rings (treated with the appropriated vehicle) were run in parallel with experimental rings. The second set of experiments was performed using nominally Ca^2+^-free medium (containing 0.1 mM EGTA to chelate trace Ca^2+^) and consisted of concentration-response curves to CaCl_2_  (3 × 10^−5^–3 × 10^−2 ^M) obtained in the absence or in the presence of vardenafil (10^−7^–10^−6 ^M) in pulmonary artery rings taken from pulmonary hypertensive group.

### 2.6. Statistical Analysis

 Experimental values of relaxation or contraction were calculated relative to the maximal changes from the contraction produced by Phe and KCl, respectively, taken as 100% in each tissue. In order to evaluate the effects of vardenafil; maximum response (*E *_max_), the contraction for a half maximal response (EC_50_), and pD_2_ values were calculated. These values were calculated from the concentration-response curve obtained in each experiment, as predicted from the Scatchard equation for drug receptor interaction. Data are shown as the percentage of relaxation of *n* experiments, expressed as the mean ± S.E.M. Data were analyzed by two-way ANOVA for multiple comparisons followed by Bonferroni post hoc test. *P* < 0.05 was considered to indicate significance. A program package was used for the statistical analysis of all data (GraphPad Instat, 1997, version 3.00; GraphPad Software Inc., San Diego, CA).

## 3. Results

### 3.1. Effects of Monocrotaline on Rat Cardiac Weight and Pulmonary Arteries Morphology

**Figure 1:**
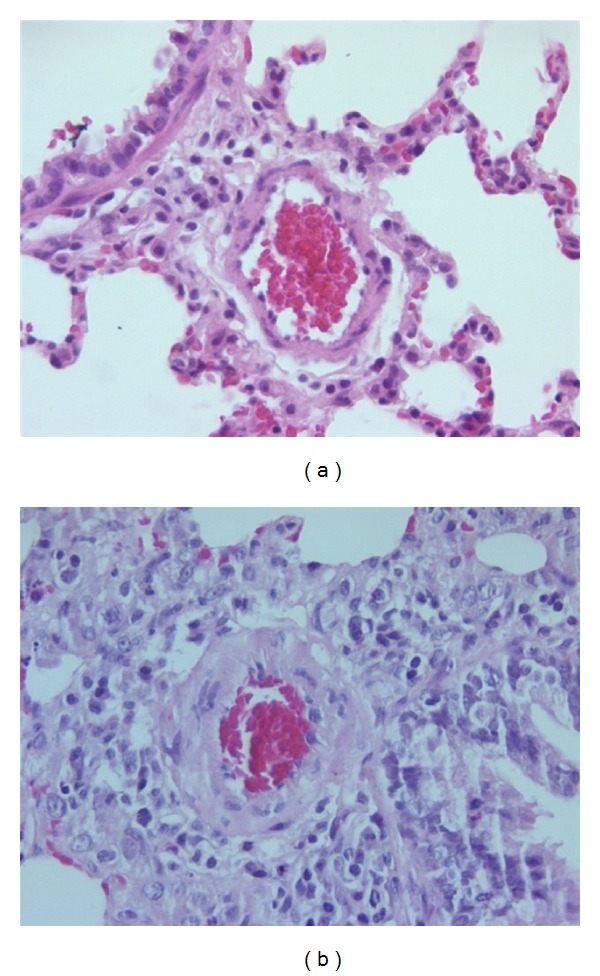
Pulmonary arteries were demonstrated in lungs from control group (a) and from the rat with monocrotaline-induced pulmonary hypertension (b).

As shown in Table 1, body weight was significantly decreased in the MCT-treated group after 21 days of administration. Moreover, the ratios of RV weight to body weight and RV weight to LV + S weight were increased in MCT-treated rats, suggesting the occurrence of right ventricular hypertrophy. Furthermore, the thickness of the medial wall (WT) and the value of WT% were significantly increased in pulmonary hypertensive group as compared with control (Table 2) (Figures 1(a) and 1(b)).

### 3.2. Vascular Reactivity Studies

**Figure 2:**
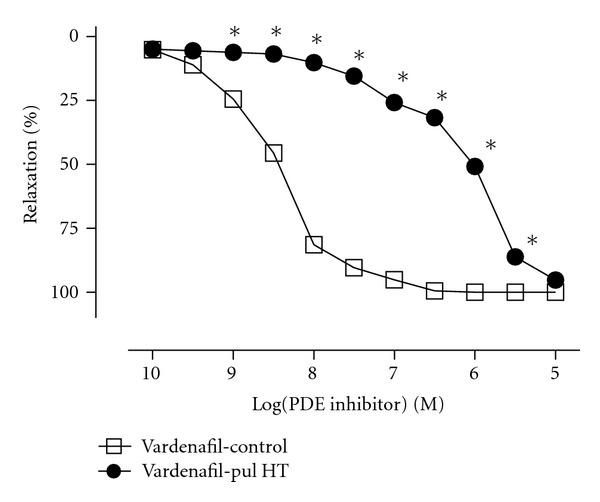
Concentration- response curves (10^−10^–10^−5^ M; *n* = 15) to vardenafil in control and pulmonary hypertensive rat pulmonary artery rings contracted by phenylephrine (10^−6^ M). Experimental values were calculated relative to the maximal changes from the contraction produced by phenylephrine in each tissue, which was taken as 100%. *P < 0.05 as compared with control.

**Figure 3:**
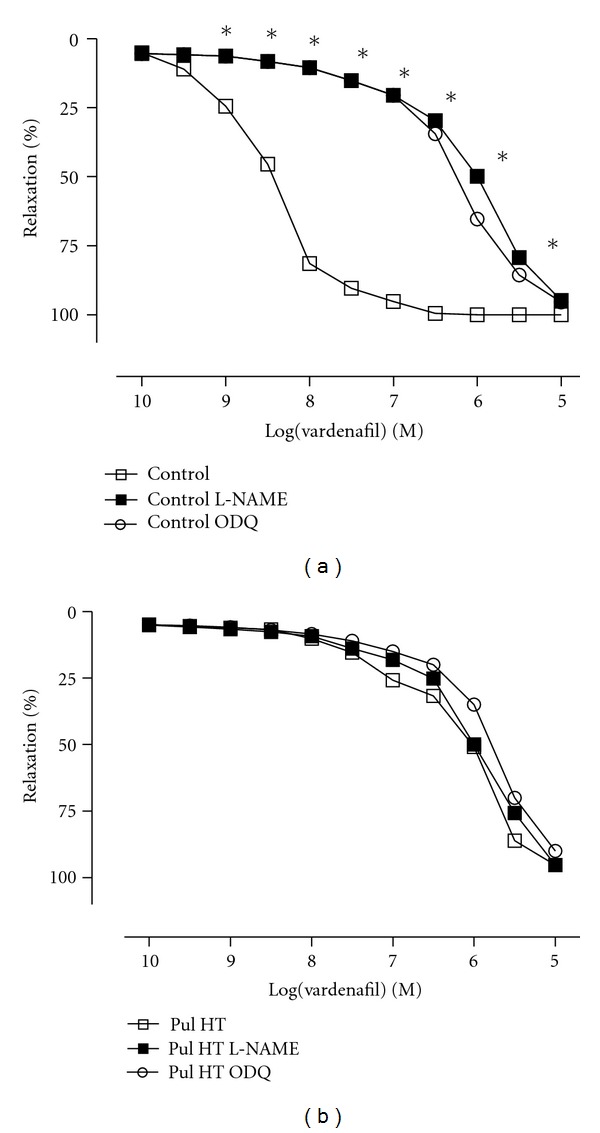
Effects of L-NAME (10^−4^ M; *n* = 15) and ODQ (10^−5^ M; *n* = 15) on the relaxations induced by vardenafil in control (a) and pulmonary hypertension group (b). Data were calculated relative to the maximal changes from the contraction produced by phenylephrine (10^−6^ M) in each ring, which was taken as 100%. *P < 0.05 as compared with control.

Vardenafil (10^−10^–10^−5^ M) induced concentration-dependent relaxation response (pD_2_  8.53 ± 0.04). In pulmonary hypertensive group, the relaxations induced by vardenafil was significantly attenuated, as evidenced by the marked rightward shifts (pD_2_  6.02 ± 0.03) (Figure 2). Interestingly, maximal response of relaxation to vardenafil was not affected by the development of pulmonary hypertension. Experiments in L-NAME (10^−4^ M) and ODQ (10^−5^ M) were used to verify the involving of the NO/cGMP pathway in PDE-5 inhibitor-induced relaxations. L-NAME or ODQ were added to the organ bath 20 min before precontraction with phenylephrine. Incubations resulted in a significant reduction in relaxation responses both in control and pulmonary hypertensive group rats and caused marked rightward shifts in the curves to vardenafil. The maximum responses of vardenafil were not significantly change in the absence or presence of these incubations (Figures 3(a) and 3(b)).

**Figure 4:**
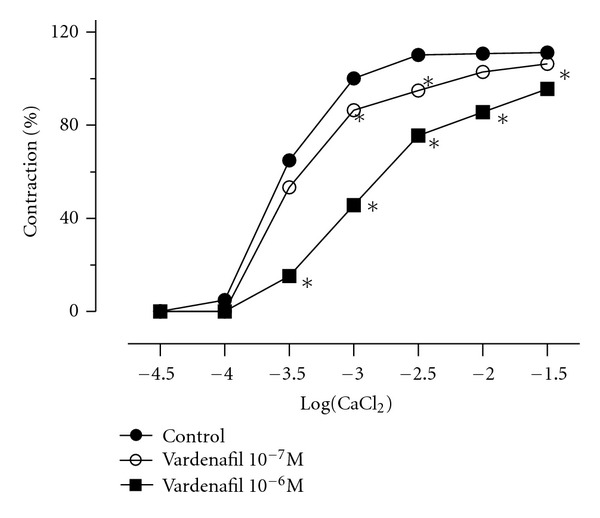
Concentration-response curves to CaCl_2_ in the absence or presence of vardenafil (10^−7^–10^−6^ M; *n* = 13) in pulmonary hypertensive rat pulmonary artery rings. Data were calculated relative to the maximal changes from the contraction produced by KCl (80 mM) in each ring, taken as 100%. *P < 0.05 as compared with control.

 The second set of experiment was performed in Ca^+2^-free Krebs solution containing EGTA. Vardenafil (10^−7^–10^−6^ M) concentration dependently inhibited contractions evoked by CaCl_2_  (3 × 10^−5^–3 × 10^−2 ^M) with reducing the maximum response to CaCl_2_ (Figure 4).

## 4. Discussion

This is the first analysis of the pharmacological profiles of specific PDE-5 inhibitor vardenafil, in the rat MCT-induced pulmonary artery hypertension model. In our study, by examining the vasorelaxant properties of vardenafil, we demonstrated that vardenafil potently caused pulmonary artery relaxation in a dose-dependent fashion through mechanisms involving both NO/cGMP-dependent and -independent pathways.

Pulmonary arterial hypertension is characterized by increased pulmonary vascular resistance, narrowing of pulmonary vascular lumen due to thickening of the vessel media, changes in functional parameters of the lung vasculature, and right ventricular hypertrophy [21]. In our study, pulmonary hypertension model was established with the development of right ventricular hypertrophy. Specifically, we used the ratio of the weight of the right ventricle (RV) to the weight of the left ventricle plus septum (RV/(LV + S)) which was found to be a reliable index of right ventricular hypertrophy in previous studies [22–24]. The present study showed that the arterial medial thickening in the pulmonary vasculature was significantly increased in the MCT group as well as the increase in right ventricular hypertrophy. In this way, pulmonary hypertension is evidenced with these morphological changes in our experimental model.

Although the underlying mechanisms of PAH have not been fully elucidated, it has been shown that vasodilator therapies including PDE-5 inhibition, can reduce the increase in pulmonary vascular resistance and cause vasodilatation. [25, 26]. When compared with expression of PDE-5 in other tissues such as myocardium, the expression and activity of PDE-5 is considerably higher in lung and pulmonary vascular smooth muscle cells [8]. Phosphodiesterase type-5 inhibitors are therefore now receiving attention for their beneficial effects in PAH. Moreover, sildenafil and tadalafil are currently approved for the treatment of PAH. In light of the foregoing, we aimed to investigate the effects of other commercially available PDE-5 inhibitor, vardenafil, in MCT-induced pulmonary artery hypertension.

Our findings confirm that vardenafil is an effective pulmonary vasodilator and the incubation with NO-cGMP pathway inhibitors (L-NAME or ODQ) attenuates the vasodilator action of vardenafil. This finding is in agreement with previous studies in that PDE-5 is implicated in the process via inactivating cGMP [27]. Besides these incubations, pulmonary hypertension also decreased the relaxation responses of vardenafil in our study, and it caused marked rightward shifts in the curves to vardenafil. But, maximal responses to vardenafil were not affected with incubations or pulmonary hypertension. Our findings indicated that vardenafil, in addition to inhibiting PDE-5 and provoking pulmonary artery relaxation via NO-cGMP-dependent mechanisms, also evokes activation of some NO-cGMP-independent mechanisms. Recently, vardenafil was shown in a twelve-week, randomized, double-blind, placebo-controlled study involving 66 patients to improve 6-minute walking distance and pulmonary hemodynamic [28]. In that study, vardenafil was associated with only mild and transient adverse events. But clinicians should be aware of the hypotension occurring risk as a class effect of PDE-5 inhibitors in combination with nitrates [29].

Calcium (Ca^+2^) influx through channels represents one of the major pathways to control the vascular tone [30]. Its blockade causes vasorelaxation or inhibits the vasocontraction. Hence, we sought to investigate effects of vardenafil on contractions induced by CaCl_2_ in Ca^+2^-free medium. At concentrations 10^−7^ and 10^−6^ M, vardenafil shifted the curves for CaCl_2_ to the right along with significant reductions in maximal responses. Our findings simply suggest that, at least in the concentrations employed in this study, vardenafil inhibits the external Ca^+2^ entry. This possibility is in agreement with previous studies demonstrating that vardenafil, but not sildenafil or tadalafil, affects Ca^+2^ handling with its Ca^+2^-channel blocking activity in the rat aorta, in addition to rabbit pulmonary artery and human washed platelets [7, 27].

In conclusion, the data reported herein show that vardenafil potently relaxes pulmonary artery rings through NO-cGMP-dependent and -independent mechanisms. In addition, vardenafil-induced relaxations seem to involve blockade of Ca^+2^ entries. The results in this suggest that vardenafil may be more effective than sildenafil and tadalafil for the treatment of pulmonary hypertension. Also, the clinical implications of this research require further randomized, placebo-controlled studies to precisely define the long-term efficacy, side effects, and drug interactions of vardenafil in order to emphasize its place in the management of PAH.

## Figures and Tables

**Table 1:** Effects of monocrotaline treatment on rat body weight and cardiac weight. Results are expressed as mean ± s.e.mean. *Significantly different from control rat at *P* < 0.05. Number of animals is indicated in parentheses. BW (0 days): body weight before injection; BW (21. day): body weight at 21 days after injection; LV + S: wet weight of left ventricle plus septum; RV, wet weight of right ventricle; RV/ LV + S: ratio of RV to LV + S.

	Control (*n* = 20)	MCT (*n* = 20)
BW (0 day, gr)	195 ± 18	170 ± 3
BW (21.day, gr)	317± 15	225 ± 3*
RV/BW (gr/kg)	0.57 ± 0.02	1.15 ± 0.03*
RV/LV + S	0.23 ± 0.01	0.59 ± 0.01*

**Table 2:** Monocrotaline-induced morphometric changes in pulmonary arteries. Results are expressed as mean ± s.e. mean. *Significantly different from control rat at *P* < 0.05. Number of animals is indicated in parentheses. WT: media wall thickness; ED: external diameter.

	Control (*n* = 15)	MCT (*n* = 16)
WT (*μ*)	9.7 ± 0.5	19.2 ± 1.2*
ED (*μ*)	84 ± 6	95 ± 5
% WT	21.1 ± 1.2	43.1 ± 1.1*
